# Depletion of Toll-Like Receptor-9 Attenuates Renal Tubulointerstitial Fibrosis After Ischemia-Reperfusion Injury

**DOI:** 10.3389/fcell.2021.641527

**Published:** 2021-02-12

**Authors:** Haofeng Zheng, Yannan Zhang, Lei Li, Rui Zhang, Zihuan Luo, Zhe Yang, Yongrong Ye, Jiannan He, Qiquan Sun

**Affiliations:** Organ Transplantation Research Institute of Sun Yat-sen University, The Third Affiliated Hospital of Sun Yat-sen University, Guangzhou, China

**Keywords:** TLR-9, acute kidney injury, chronic kidney disease, fibrosis, macrophage

## Abstract

Toll-like receptor-9 (TLR-9) is a potent proinflammatory receptor that mediates renal injury. However, the reported effects of TLR-9 are contradictory. Here, using a traditional mouse AKI→CKD transition model, the roles of TLR-9 during the transition from acute kidney injury (AKI) to chronic kidney disease (CKD) were further explored. Using a TLR-9^–/–^ mouse, the effects and mechanisms of TLR-9 were investigated. Loss of TLR-9 elicited no obvious effects as regards renal function or histology during AKI in the early phases (24–48 h), while TLR-9 KO attenuated renal fibrosis (as shown using fibronectin and collagen III) and epithelial-to-mesenchymal transition (EMT) [E-cadherin (E-Cad) and α-smooth muscle actin (α-SMA)] on the long-term after AKI through the inhibition of macrophages infiltration, especially M2 macrophages. The roles of TLR-9 on macrophages were also explored using Raw264.7 macrophage cell line, and results indicated that the inhibition of TLR-9 on Raw 264.7 macrophages decreased the induction of M2 type macrophage in a dose-dependent manner. The roles of TLR-9 on renal tubular epithelial (RTE) cells were also explored. Conversely, TLR-9 depletion did not contribute to the improvement of fibrosis and EMT *in vitro*. Therefore, TLR-9 plays a critical role in the AKI→CKD transition. Attenuation of CKD post-AKI in the TLR-9 KO group mainly relies on the effects of TLR-9 on macrophages. These results also suggest that TLR-9 could be a therapeutic target for CKD.

## Introduction

Acute kidney injury (AKI), first acknowledged as a syndrome over 70 years ago, primarily occurs due to ischemia-reperfusion injury (IRI), which is generally observed in renal allografts or following vascular surgery (Shu et al., 2018; Kishi et al., 2019; Li et al., 2019). AKI not only is an acute injury but also contributes to the fibrosis of the kidney. Around 10% of individuals develop chronic kidney disease (CKD) consequent to AKI worldwide, and approximately 13% of hospitalized patients experience AKI in the United States annually (Bonventre and Yang, 2011; Palomba et al., 2017). Although the natural history and etiology of AKI and CKD differ, studies have shown that AKI increases the risk of CKD by 8.8-fold, while also increasing the risk of end-stage renal disease requiring dialysis or transplantation by 3.3-fold (Coca et al., 2012).

The pathogenesis underlying the progression from AKI to CKD is complicated. Mild renal injury results in complete functional recovery, whereas more severe injury leads to CKD (Humphreys et al., 2008; Yang et al., 2010). The development of CKD from AKI can mainly be classified into four overlapping phases: priming, activation, execution, and progression. Inflammation following AKI sets up the initial conditions for CKD (priming) (Liu, 2011). Specifically, the activation of inflammatory cells, especially macrophages, is vital for the initiation and progression of CKD, whereas renal fibrosis can be attenuated by the inhibition of inflammation pathways (Su et al., 2020).

Toll-like receptor-9 (TLR-9) is identified as a receptor for bacterial unmethylated CpG-containing DNA and can lead to immune systems activation. In a cecal ligation and puncture model of sepsis, knockout (KO) of TLR-9 attenuated renal injury and reduced leukocyte migration (Tsuji et al., 2016), suggesting that TLR-9 depletion may facilitate the inhibition of inflammation. In comparison, TLR-9 was overexpressed in the tubulointerstitial compartment in patients with lupus nephritis, and TLR-9 depletion exacerbated autoimmune kidney diseases (Christensen et al., 2006; Majer et al., 2019). TLR-9 has been shown to be involved in AKI development, although reports on the detailed effects of TLR-9 in AKI remain contradictory. Moreover, although the association between the TLR-9 gene and CKD has been confirmed by several studies (Holla et al., 2010; Lu et al., 2011; Yang et al., 2013), how TLR-9 affects CKD progression remains a mystery.

To further explore the roles of TLR-9 in kidney diseases, especially chronic diseases, in this study, we utilized a traditional AKI→CKD transition rodent model to further explore the biological effects of TLR-9 on renal disease. We hypothesized that TLR-9 KO can significantly reduce renal injury and fibrosis. Epithelial-to-mesenchymal transition (EMT), leukocyte infiltrations, and inflammatory responses were explored in this study to provide more evidence for TLR-9 mechanisms during kidney disease.

## Materials and Methods

### Animals and Ethics Statement

This study was performed in accordance with the animal welfare guidelines in China (Laboratory Animal Guidelines for Ethical Review of Animal Welfare, GB/T 35892-2018) and approved by the Institutional Animal Care and Use Committee of Sun Yat-sen University. Wild-type C57BL/6J mice were obtained from Charles River Laboratories (Beijing, China), and a global TLR-9 deficient (TLR-9^–/–^) mouse was a gift from S. G. Wan (Gannan Medical University). TLR-9^–/–^ mice were backcrossed at least 10 times onto the C57BL/6J strain. Mice were genotyped using polymerase chain reaction (PCR)-based methods on DNA isolated from blood with the following primer pairs: TLR-9, Forward 1: 5′-CTG ACT TCG TCC ACC TGTC-3′, Reverse 1: 5′- TCT TCA GGG GTG GCT TCT G-3′ and Reverse 2: 5′-TTC TTG TAG TAG CAG TTC CCG-3′. All mice were housed with 12-h light/dark cycles, with normal food provided *ad libitum*. Only male mice aged 6–8 weeks were used in the subsequent experiments.

### Induction of IRI in Mice

Ischemia-reperfusion injury was induced in male mice using an established method with some modifications (Both renal pedicles were clamped for 26 min) (Yan et al., 2020). Briefly, animals were given free access to food and water 12 h before surgery. Wild-type (WT) and KO mice were anesthetized using isoflurane (5% induction and 1% maintenance) and placed on a warm pad to maintain body temperature at 36°C. Mice were randomized to the IRI or sham group (Detail number of mice per group could be found in the related paragraph). For the IRI group, the bilateral IRI (bIRI) model was used. Following midline abdominal incision, both renal pedicles were clamped using non-traumatic clamps (FT722T, Aesculap, Tuttlingen, Germany) for 28 min. Sham group mice were subjected to abdominal incision and renal pedicles isolation except for clamping of renal pedicles. Mice were euthanized using isoflurane at pre-surgery or days 1, 2, 7, 14, 21, or 28 post-surgery. Renal and blood samples were collected for subsequent analysis.

### Measurement of Renal Function

Mice were anesthetized using isoflurane (5% induction and 1% maintenance), and blood samples were collected from inferior venae cavae without any anticoagulant. Blood samples were first centrifuged at 2,000 *g* for 10 min at 4°C and then at 8,000 *g* for 10 min at 4°C. Serum was collected and frozen at −80°C until use. Serum creatinine (Cr) and blood urea nitrogen (BUN) were measured by an automatic biochemistry analyzer (7020; Hitachi, Tokyo, Japan).

### Renal Histology and Fibrosis Assessment

Renal tissues were retrieved without perfusion and fixed in 4% paraformaldehyde. Kidney paraffin sections (4 μm) were stained with hematoxylin and eosin (H&E) and periodic acid–Schiff (PAS) to assess renal injury. Sirius red (SR) and Masson’s trichrome (MT) stains were used for fibrosis assessment. Renal tubular damage assessment was performed by two independent investigators as described by Yang et al. (2018) using a score range of 0–5 (normal to extensive injury).

### Immunohistochemistry and Immunofluorescence

Renal tissues were first perfused with cold saline (4°C,0.9%), followed by 4% paraformaldehyde. Paraffin-embedded slices (4 μm) were subjected to immunohistochemistry staining, as described in our previous study (Liao et al., 2019). Briefly, sections were cultured with primary antibodies overnight at 4°C after hydrogen peroxide solution blocking (for immunohistochemistry) or non-specify antigen blocking (for immunofluorescence). Secondary antibodies, including Alexa Fluor-conjugated antibodies (Life Technologies) or HRP-conjugated antibodies, were used to detect specific antigens on the next day. Primary antibodies used in this study are listed as follows. An antibody against kidney injury molecule-1 (KIM-1, NBP1-76701; RRID:AB_11037459; Novus Biologicals, Littleton, CO, United States) was used for renal injury assessment. Antibodies against fibronectin (FN; Ab2413; RRID:AB_2262874; Abcam), collagen I (COL I, Ab88147; RRID:AB_2081873; Abcam), collagen III (COL III, NB100-92162; RRID:AB_1216519; Novus), α-smooth muscle actin (α-SMA; Ab32575; RRID:AB_722538; Abcam), and E-cadherin (E-Cad, Ab76319; RRID: AB_2076796; Abcam) were used for fibrosis and EMT assessment. An anti-TLR-9 antibody (Ab53396; RRID:AB_883065; Abcam) was used for the detection of TLR-9. Anti-F4/80 antibody (Ab6640; RRID:AB_1140040; Abcam) was used for the detection of macrophages.

### Real-Time Quantitative PCR

Fresh tissue samples were homogenized, and total RNA of tissues and monolayers were acquired using the RNAeasy^TM^ Animal RNA Isolation Kit with Spin Column (R0024; Beyotime, Shanghai, China) according to the manufacturer’s guidelines. Transcript-specific primers were generated from GenBank, and primer specificity was verified using NCBI Primer Blast. Detailed primer sequences are shown in Table 1. For Real-Time qPCR, a total of 2 μg RNA was used for reverse transcription using PrimeScript^TM^ RT master mix (RR036A; Takara, Shiga, Japan). Real-Time qPCR amplification was performed using the SYBR Green Master Mix (Roche; Bruxelles; Belgium) and the LightCycler480 system (Roche). Mean fold changes were calculated by averaging the three duplicate measurements and normalized to *Gapdh*. The 2^–ΔΔCT^ method was used for calculation.

**TABLE 1:** Primer sequences for real-time quantitative PCR.

Transcript	Seq ID	Forward 5′–3′	Reverse 5′–3′
IL-1β	NM_008361	GAAATGCCACCTTTTGA CAGTG	TGGATGCTCTCATCAGG ACAG
IL-6	NM_031168	TAGTCCTTCCTACCCCA ATTTCC	TTGGTCCTTAGCCACTC CTTC
TNF-α	NM_013693	CCCTCACACTCAGATCA TCTTCT	GCTACGACGTGGGCTA CAG
TGF-β1	NM_011577	CTCCCGTGGCTTCTAG TGC	GCCTTAGTTTGGACAGG ATCTG
CD86	NM_019388	TGTTTCCGTGGAGACG CAAG	TTGAGCCTTTGTAAATG GGCA
CD206	NM_008625	CTCTGTTCAGCTATTGA GCGC	CGGAATTTCTGGGATTC AGCTTC
IL-4	NM_021283	GGTCTCAACCCCCAGC TAGT	GCCGATGATCTCTCTCA AGTGAT
IL-10	NM_010548	GCTCTTACTGACTGGCA TGAG	CGCAGCTCTAGGAGCA TGTG
MIP-2	/	TTCCTGCTGTTTCTCTTA CACCT	CTGTCTGCCTCTTTTGG TCAG
MCP-1	NM_011333	ACCTGCTGCTACTCATT CAC	TTGAGGTGGTTGTGGA AAAG
Arg-1	NM_007482	AGGAGCTGTCATTAGG GACATC	CTCCAAGCCAAAGTCC TTAGAG
Icam-1	NM_010493	TGTTTCCTGCCTCTGA AGC	CTTCGTTTGTGATCCTC CG
Vcam-1	NM_011693	AGTTGGGGATTCGGTT GTTCT	CCCCTCATTCCTTACCA CCC
Smad 1	NM_008539	GCTTCGTGAAGGGTTG GGG	CGGATGAAATAGGATTG TGGGG
MyD88	NM_010851	TCATGTTCTCCATACCC TTGGT	AAACTGCGAGTGGGGT CAG
Snail 1	/	CACACGCTGCCTTGTG TCT	GGTCAGCAAAAGCACG GTT
TLR-9	NM_031178	CCAGTTTGTCAGAGGG AGCC	GGACAGGTGGACGAAG TCAG
Gapdh	NM_008085	AATGGATTTGGACGCA TTGGT	TTTGCACTGGTACGTGT TGAT

### Flow Cytometry

Flow cytometry was performed as previously described (Zhao et al., 2018). Briefly, kidneys were weighed and minced. A collagenase solution (1 mg/ml; Sigma-Aldrich, St. Louis, MO, United States) was used for renal digestion for 30 min at 37°C. A 100-μm cell strainer (Fisher Scientific) was used, together with a 1-ml syringe plunger, to acquire single cells. Cell pellets were washed and resuspended for further experiments. Anti-Mouse CD16/CD32 (553141; RRID:AB_394656, clone 2.4G2; BD Biosciences, San Jose, CA, United States) was used for non-specific Fc block. Antibodies used in this assay were acquired from BioLegend, San Diego, CA, United States; they include BV421 anti-mouse CD45 (RRID:AB_2562559), APC anti-mouse F4/80 antibody (RRID:AB_893481), PE anti-mouse CD206 (MMR) antibody (RRID:AB_10895754), FITC anti-mouse CD86 antibody (RRID:AB_313149), PerCP/Cyanine5.5 anti-mouse/human CD11b (RRID:AB_893232), APC/Cyanine7 anti-mouse CD3 antibody (RRID:AB_2242784), FITC anti-mouse CD4 antibody (RRID:AB_312713), and APC anti-mouse CD8a antibody (RRID:AB_312750). Data were acquired on a FACS Calibur cytometer [Becton Dickinson (BD), Bedford, MA, United States] and analyzed using FlowJo software (Tree Star, Ashland, OR, United States).

### Cell Culture

The Raw 264.7 murine macrophage was obtained from the ATCC (Bethesda, MD, United States). Cells were cultured in RPMI 1,640 supplemented with 10% of FBS (Gibco, United States), streptomycin (100 μg/ml, Life Technologies), and penicillin (100 units/ml, Life Technologies) and incubated overnight at 37°C and 5% CO_2_.

### Isolation of Primary RTE Cells

Renal tubular epithelial (RTE) cells (a mixture of RTE cells) were extracted from adult male mice (6–8 weeks of age) following the method described by Ichimura et al. (2008). A collagenase solution (1 mg/ml; Sigma-Aldrich, St. Louis, MO, United States) was used for renal digestion. Primary tubules were resuspended in tubule medium consisting of Dulbecco’s modified Eagle medium: F12 culture medium (Life Technologies, Carlsbad, CA, United States), epidermal growth factor (10 ng/ml; Sigma-Aldrich), hydrocortisone (50 nM; Selleck Chemicals, Houston, TX, United States), tri-iodothyronine (32 ng/ml; Selleck), insulin/transferrin/selenium (10 μg/ml/5.5 μg/ml/5 ng/ml; Life Technologies), and 1% penicillin–streptomycin (Life Technologies). Plates for cell culture were treated with collagen I (Corning, Armonk, NY, United States) before cell culture. RTE cells were isolated, and immunofluorescence of anti-cytokeratin 18 antibody (CK18; Ab668; RRID:AB_305647; Abcam) was used to verify the phenotype of epithelial cells (Supplementary Figure 1). The second passage of RTE cells was used for subsequent assays. The plating density of RTE cells varied depending on the type of plates, 1 × 10^6^ for 6-well plates and 1 × 10^5^ for 48-well plates.

### Effects of ODN2088 on the Differentiation of Raw264.7 Cell Lines

As for differentiation of Raw264.7 mouse macrophages, Raw264.7 were stimulated for IL-4 (20 ng/ml, Novoprotein, China) and IL-13 (20 ng/ml, Novoprotein, China) or LPS (250 ng/ml, Sigma) and IFN-γ (20 ng/ml, Sigma) for 24 h. TLR9 antagonist CpG-oligodeoxynucleotides (ODN) (2088; Invivogen, San Diego, CA, United States) or control CpG-ODN (Invivogen) was co-cultured with stimulants to determine the effects of TLR-9 on macrophages.

### Cellular Immunofluorescence

For cellular immunofluorescence, cells were washed three times with phosphate-buffered saline (Sigma) to remove the culture medium. Fixation and permeabilization were performed for 15 min with 4% paraformaldehyde (P0099, Beyotime) and 20 min with 1% Triton X-100 (P0096, Beyotime). QuickBlock^TM^ Blocking Buffer for Immunol Staining (P0260, Beyotime) was used for non-specific antigen blockage. Primary antibodies were used according to the goals of each experiment and incubated overnight. Secondary antibodies, including Alexa Fluor-conjugated antibodies (Life Technologies), were used to detect specific antigens the next day. Nuclear staining was performed using 4′,6-diamidino-2-phenylindole (DAPI; P0131; Beyotime).

### Induction of Hypoxia-Reoxygenation Injury in RTE Cells *in vitro*

Renal tubular epithelial cells were grown in a normal medium until reaching 80% confluence in a normal oxygen incubator (21% O_2_, 5% CO_2_, and 74% N_2_) and then cultured under hypoxic conditions (1% O_2_, 5% CO_2_, and 94% N_2_) for 24 h. RTE cells were then reoxygenated to normal oxygen for 1 h. RNA was acquired as described above; cellular immunofluorescence was also performed.

### Histology, Immunohistochemistry, and Immunofluorescence Analysis

Quantification of fibrosis, immunohistochemistry, and immunofluorescence analysis results was performed using Image J (NIH Image, Bethesda, MD, United States). Five randomly chosen high-resolution images per mouse (original magnification x200) captured using the Leica system were used for quantification. Two independent investigators blinded to experimental conditions performed this process.

### Statistics

The KS normality test was first used to test whether the data were normally distributed; all data met this criterion. The Student’s *t*-test (2 groups) and one-way analysis of variance (≥3 groups) were used to analyze significant differences. All values are expressed as the mean ± standard deviation (SD). Data were analyzed using GraphPad Prism 8.0 (GraphPad Software Inc., La Jolla, CA, United States). *P*-values < 0.05 were considered statistically significant.

## Results

### TLR-9 Is Redundant for Early Renal Recovery Following IRI

**FIGURE 1:**
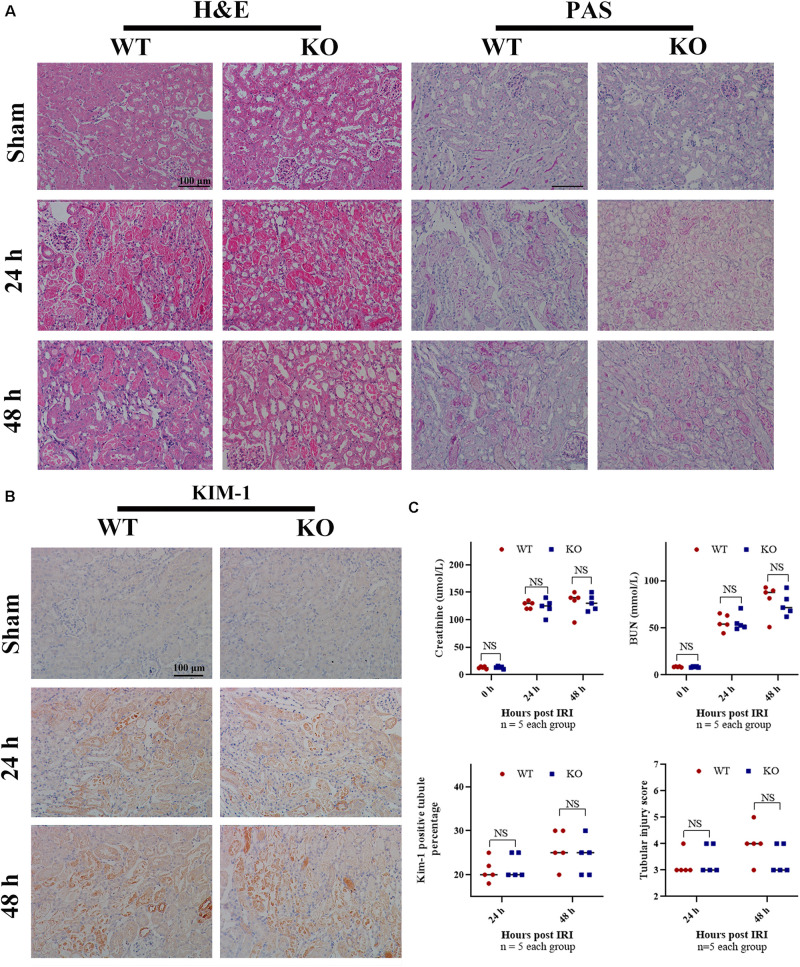
TLR-9 depletion has no obvious effect on the recovery of renal function and injury following IRI in the early phases. WT and TLR-9 KO mice were exposed to 26 min of bIRI or sham surgery. Renal tissues and sera were acquired 24 or 48 h post-injury. Tissue sections are representative of five mice per group. **(A)** Representative sections of the renal outer medulla stained with H&E and PAS post IRI or in the sham group. **(B)** Representative KIM-1 immunohistochemistry in renal cortical sections from WT and KO mice at day 0, 1, or 2 post-IRI. **(C)** Upper panel: serum creatinine and urea from WT and KO mice at day 1 or 2 following renal IRI or in the sham group; lower panel: semiquantitative analysis of tubular damage in kidneys from WT and KO mice following IRI and semiquantitative analysis of KIM-1 immunohistochemistry staining. Data are shown as the means ± SD and analyzed by using the Student’s *t* test. Magnification: 200×. NS, no significant difference; WT, wild-type; KO, knockout; H&E, hematoxylin and eosin; PAS, periodic acid–Schiff.

To determine whether TLR-9 participates in acute renal IRI, serum Cr and BUN of WT and KO mice were assessed 24 and 48 h post-IRI. It seemed that tubular casts on the KO mice were less compared to those on WT mice, while no significant difference was found regarding serum Cr and BUN between KO sham mice and WT sham mice (Figures 1A,C). In contrast to the hypothesis of this study, TLR-9 depletion did not attenuate early renal function, based on Cr and BUN, following IRI. Kidneys from both groups subjected to IRI exhibited obvious tubular injury, including cast formation, loss of the brush border, tubular dilation, and extensive loss of tubular epithelial cells (Figure 1A). No significant difference was found regarding the levels of serum Cr or BUN between TLR-9 KO and WT mice or the renal tubular injury scores at 24 and 48 h (Figure 1C). Expression of KIM-1, a biomarker for renal injury, was barely detectable in the two groups that underwent sham injury but increased 24 h post-IRI. Nevertheless, no significant difference was identified between the TLR-9 KO and WT groups (*P* (>0.05) (Figures 1B,C).

### TLR-9 Deficiency Reduces Renal Tubulointerstitial Fibrosis Following IRI

**FIGURE 2:**
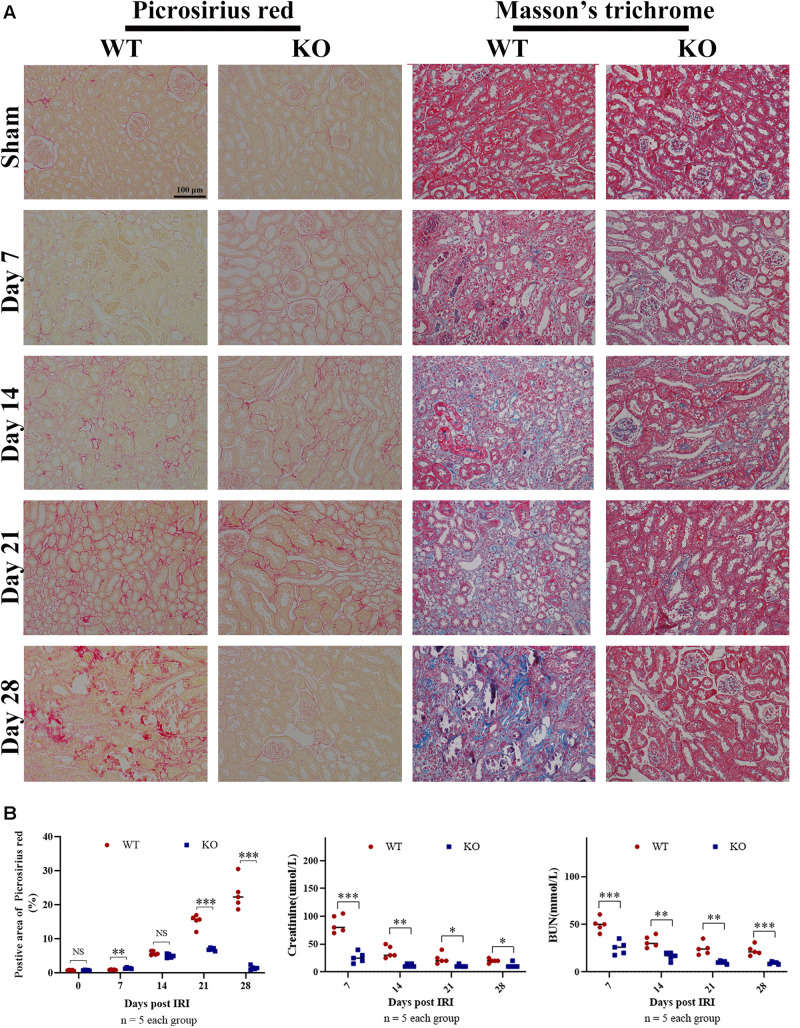
TLR-9 deficiency reduces renal tubulointerstitial fibrosis following IRI. Mice in WT and KO groups were subjected to IRI. Serum samples and kidneys were collected at 7, 14, 21, and 28 days following reperfusion. Tissue sections are representative of five mice per group. **(A)** Representative images of Sirius red and Masson’s trichrome stains on normal and injured kidneys of WT and KO mice at various time points following reperfusion. **(B)** Left panel: quantification of Sirius red-stained areas in murine renal cortical medullary junction sections; middle and right panel: serum creatinine and urea from WT and KO mice at various time points following reperfusion. Data are shown as the means ± SD and analyzed using the Student’s *t* test. Magnification: 200×. *P < 0.05, **P < 0.01, and ***P < 0.001. NS, no significant difference; IRI, ischemia-reperfusion injury; WT, wild-type; KO, knockout.

**FIGURE 3:**
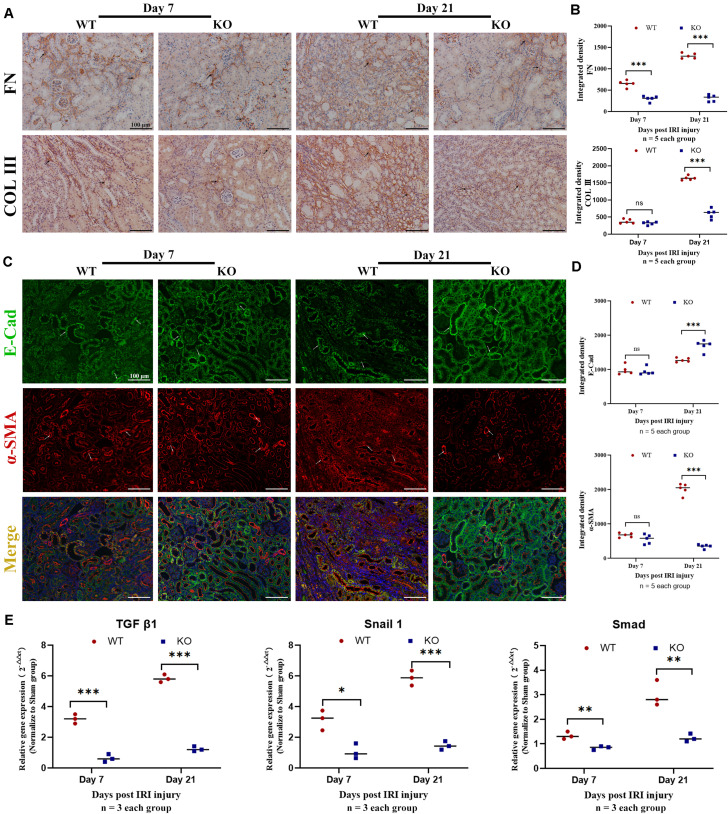
Knockout of TLR-9 attenuates EMT and inhibits profibrotic factors following IRI. Renal tissues were acquired at 7 and 21 days following reperfusion in WT and KO mice. Tissue sections are representative of five mice per group. **(A)** Representative COL III and FN staining immunohistochemistry in renal cortical sections from WT and KO mice at day 7 or 21 post-IRI. **(B)** Semiquantitative analysis of COL III, and FN immunofluorescence staining of WT and KO mice at various time points following reperfusion. **(C)** Representative fluorescence microscope images of E-Cad (green) and α-SMA (red) staining of the kidneys of WT and KO mice at various time points following reperfusion; **(D)** Semiquantitative analysis of E-Cad and α-SMA immunofluorescence staining of WT and KO mice at various time points following reperfusion. **(E)** Expression of profibrotic factors in WT and KO mice at various time points following reperfusion. The arrows indicate positive stained signals. Data are shown as the means ± SD and were analyzed by the Student’s *t* test. Each dot represents an individual mouse. Magnification: 200×. *P < 0.05, **P < 0.01, and ***P < 0.001. NS, no significant difference; EMT, epithelial-to-mesenchymal transition; RTE, renal tubular epithelial; TLR-9, toll-like receptor-9; IRI, ischemia-reperfusion injury; WT, wild-type; KO, knockout; E-Cad, E-cadherin; α-SMA, α-smooth muscle; COL I, collagen I; FN: fibronectin.

Time-kinetics of the development of fibrosis was observed in KO and WT mice. Renal tissues were obtained at 7, 14, 21, and 28 days post bIRI. Tubulointerstitial fibrosis was assessed using MT and SR staining (Figures 2A,B). The degree of tubulointerstitial MT- and SR-positively stained area increased gradually over time in the WT group, whereas the area was significantly reduced in KO mice (*P* < 0.01). Renal function measures, including BUN and SCr, were also monitored during disease progression. Renal function at days 7 and 14 was better preserved in KO mice, whereas the difference was less apparent by day 21 post-IRI (Figure 2B). Histology assessment using H&E and PAS showed that TLR-9 depletion could contribute to renal function recovery following AKI injury, as kidneys in the KO group presented with less leukocyte infiltration injury than those in the WT group (data not shown). Immunohistochemistry was also applied to evaluate the distribution of FN and COL III to assist with the assessment of fibrosis. No significant difference was found between the WT sham group and the KO sham group regarding FN or COL III (Supplementary Figure 2). Expression of FN and COL III was significantly inhibited in the KO group than in the WT group (*P* < 0.01) (Figures 3A,B).

### TLR-9 Deficiency Attenuates EMT Following IRI

Epithelial-to-mesenchymal transition, which leads to renal epithelial cell dedifferentiation and promotes fibrosis, plays an important role in renal fibrosis. Since EMT could be regulated by inflammation and TLR-9 is involved in multiple inflammation processes, the roles of TLR-9 in EMT were also explored. Immunohistochemistry for EMT, including E-Cad (an epithelial marker) and α-SMA (indicating a subset of activated fibrogenic cells), was therefore performed. No significant difference was found between the WT sham group and the KO sham group regarding E-Cad or α-SMA (Supplementary Figure 2). The expression of E-Cad was significantly higher in KO than WT mice 21 days post-IRI (*P* < 0.01), whereas the expression of α-SMA was lower in KO mice (*P* < 0.01) (Figures 3C,D). Profibrotic factors, including TGF-β, snail 1, and Smad, were also assessed using RT-qPCR; it was revealed that TLR-9 KO inhibited profibrotic factors (Figure 3E).

### TLR-9 Deficiency Decreases the Number of Leukocyte and Macrophage in the Kidney Following IRI

**FIGURE 4:**
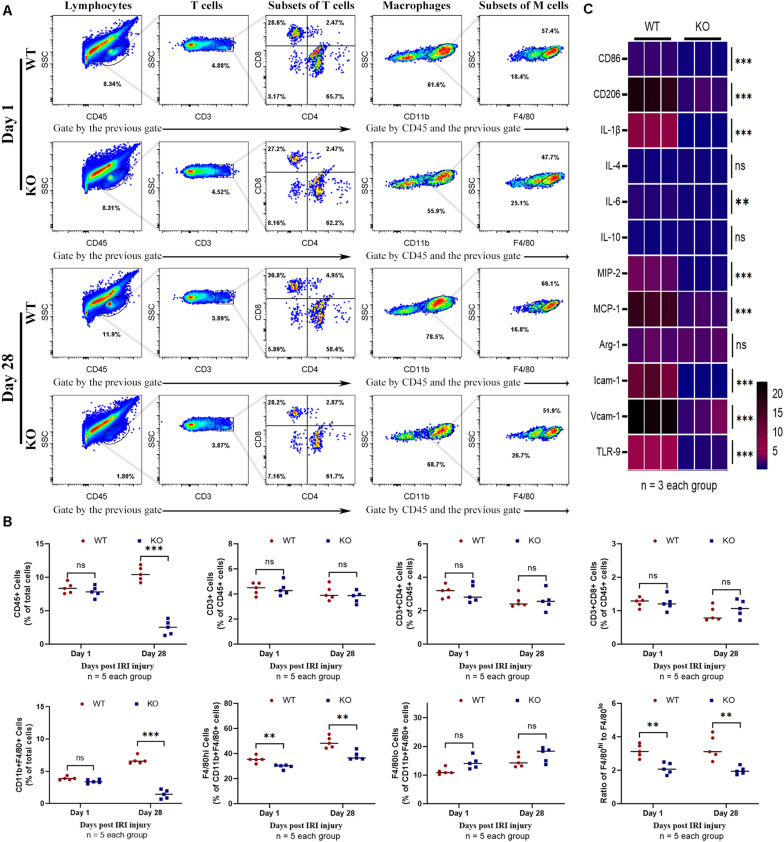
Effect of TLR-9 deficiency on leukocytes infiltration and inflammation related cytokines in the kidney following renal IRI. Inflammatory macrophage infiltration in WT and KO kidneys at 1 and 28 days following IRI were determined using flow cytometry. **(A)** Representative dot plots of CD45+ cells and different types of leukocytes (T cells and macrophages) of WT and KO mice at various time points following reperfusion. **(B)** Quantification of related immune cell infiltration, including CD 45+ cells, CD3+ T cells, F4/80^high^, and F4/80^low^ subset in CD11b+. **(C)** Heatmap of mRNA expressions related to inflammation in renal 28 days post IRI. Data are shown as the mean ± SD or percentage and analyzed using the Student’s *t*-test. *P < 0.05, **P < 0.01, and ***P < 0.001. NS, no significant difference; IRI, ischemia-reperfusion injury; WT, wild-type; KO, knockout.

Leukocyte infiltration constitutes an important hallmark of inflammation in tissue injury. The number of leukocytes, especially macrophages that substantively contribute to IRI, was observed using flow cytometry. Results showed that KO mice exhibited significantly fewer CD45+ (total leukocyte) cells 1 and 28 days post-IRI (Figure 4A). Fewer macrophages were observed in KO than WT mice 28 days post-injury (*P* < 0.01) (Figures 4A,B). The phenotypes of macrophages in the kidney were further analyzed. Although M1-like phenotype (CD11b+, F4/80^low^) and M2-like phenotype (CD11b+, F4/80^high^) macrophages were inhibited in the KO group; it was found that M2-like phenotype decreased more than M1-like phenotype macrophages post-bIRI compared to the WT group (*P* < 0.01) (Figures 4A,B). Infiltrations of T cell (CD3+) and its subsets were also detected in this assay. No significant difference was found between the WT and KO groups regarding T cells 1 or 28 days post-bIRI (Figures 4A,B). Chemokine production, which is related to renal inflammation, 28 days post-IRI was also explored. The results indicated that TLR-9 KO decreased the expression of multiple pro-inflammation cytokines, including CD86, CD206, IL-1β, IL-6, MIP-2, MCP-1, Icam-1, and Vcam-1 (Figure 4C).

### TLR-9 Affects Induction of Raw264.7

**FIGURE 5:**
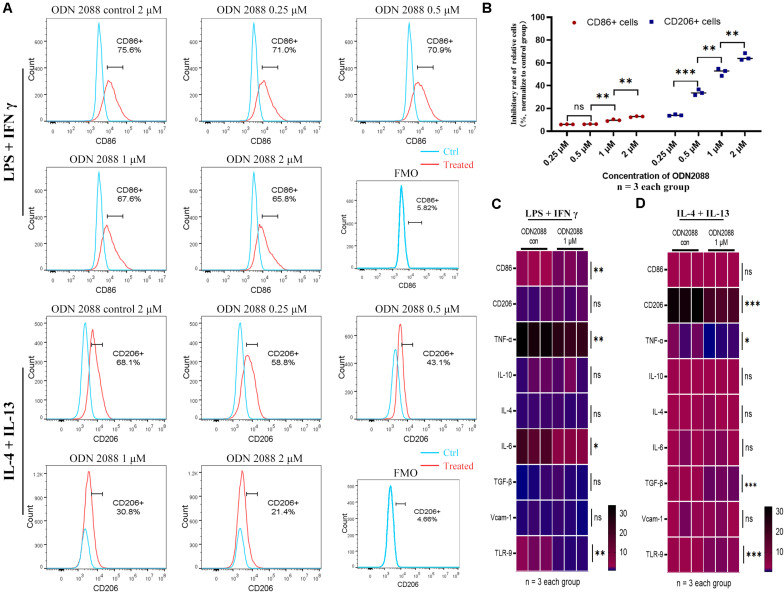
Inhibition of TLR-9 affects differentiation and function of Raw264.7 *in vitro*. **(A)** Effects of ODN2088 on macrophage polarization. **(B)** Quantitative analysis of inhibition rate for macrophage subsets. **(C)** Heatmap of mRNA expressions related to macrophage polarization when stimulated with LPS + IFN-γ. **(D)** Heatmap of mRNA expressions related to macrophage polarization when stimulated with IL-4 + IL-13. Raw264.7 was polarized into M1 or M2 subset, and various concentrations of ODN2088 were added for 24 h to assess the effects of TLR-9 antagonist on differentiation of macrophages. Data is shown as the mean and analyzed using the Student’s *t*-test. NS, no significant difference. *P < 0.05, **P < 0.01, and ***P < 0.001.

To further confirm the effects of TLR-9 on macrophages, a mouse macrophage cell line Raw264.7 and a TLR-9 antagonist (ODN2088) were used. TLR-9 was up-regulated post stimulation with LPS and IFN-γ or IL-4 and IL-13 and down-regulated post ODN2088 culture. A high dose of ODN2088 (2 μM) inhibited the induction of M1 and M2 macrophages, while a limited effect of low dose ODN2088 on the M1 subset was found. The results showed that inhibition of TLR-9 decreased more M2-like macrophages than M1-like macrophages (Figures 5A,B). Chemokine production and cytokines of Raw264.7 post induction were also explored. Inhibition of TLR-9 decreased the expressions of CD86, TNF-α, and IL-6 when stimulated with LPS and IFN-γ, while CD206, TNF-α, and TGF-β were decreased when stimulated with IL-4 and IL-13 (Figures 5C,D).

### Depletion of TLR-9 Did Not Affect Macrophage-to-Myofibroblast Transition

**FIGURE 6:**
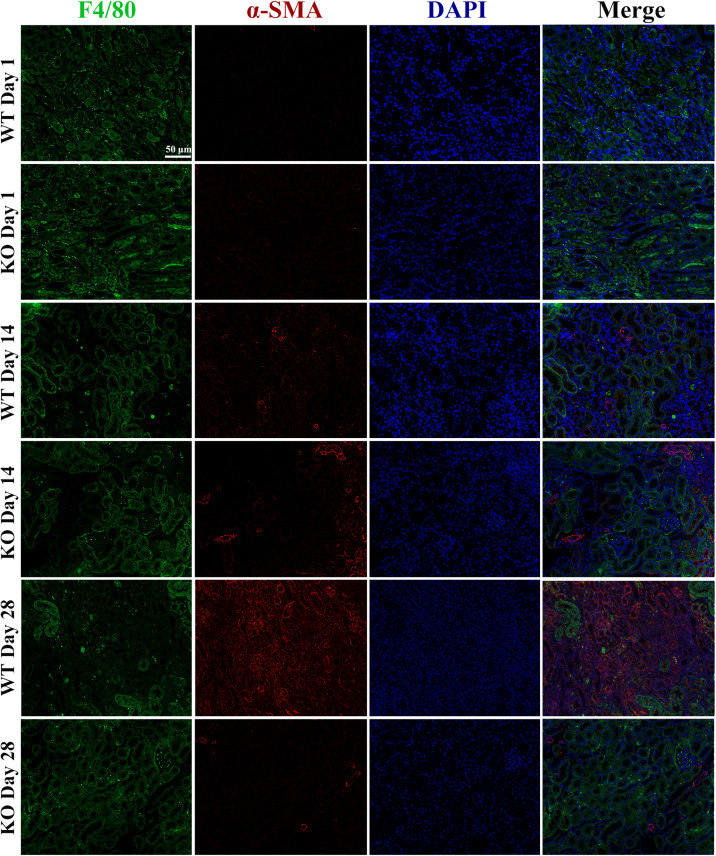
Depletion of TLR-9 has no effects on macrophage-to-myofibroblast transition. Renal tissues were acquired at 1, 14, and 28 days following reperfusion in WT and KO mice. Tissue sections are representative of five mice per group. Immunofluorescence of F4/80 and α-SMA was used to assess macrophage-to-myofibroblast transition.

Considering the reasons why TLR-9 is mainly expressed on macrophages and why macrophages could promote fibrosis through macrophage-to-myofibroblast transition (MMT), the effects of TLR-9 on MMT were also explored (as determined by F4/80 and α-SMA). Renal tissues of the WT and KO groups were acquired on days 1, 14, or 28 post-bIRI. Cells that expressed both F4/80 and α-SMA could not be detected (Figure 6), indicating that the effects of TLR-9 on MMT seem limited.

### Depletion of TLR-9 Did Not Attenuate EMT of RTE Cells *in vitro*

**FIGURE 7:**
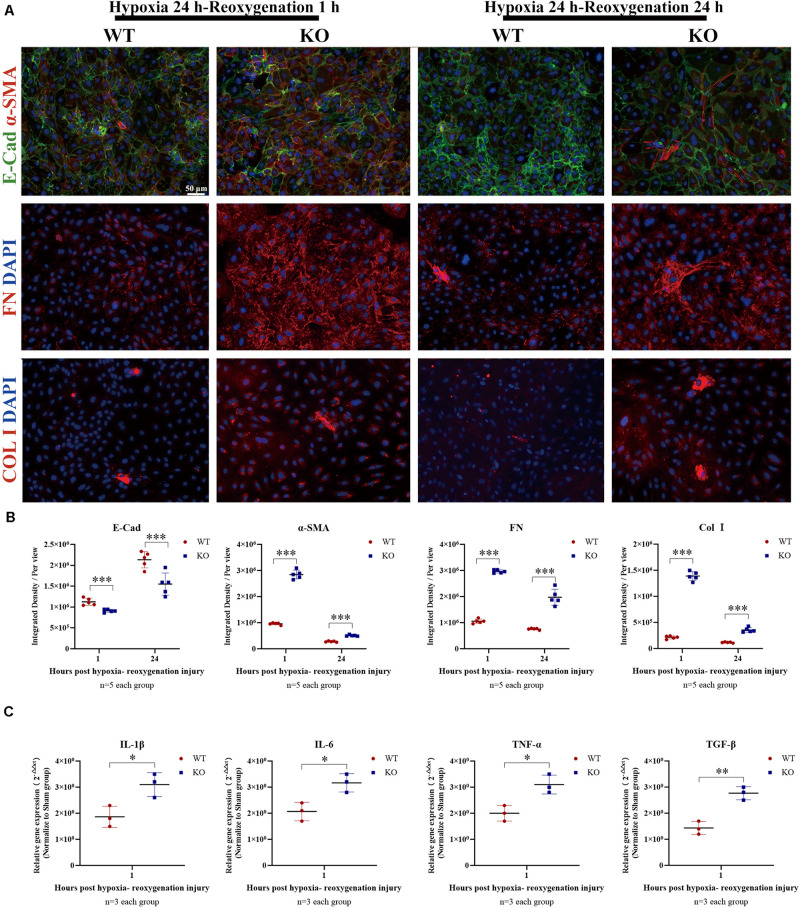
Depletion of TLR-9 didn’t attenuate EMT of RTE cells *in vitro*. RTE cells isolated from WT and KO mice were exposed to 24-h hypoxia and then reoxygenated in normal oxygen for 1 h. Results were acquired from three independent assays. **(A)** Immunofluorescence of COL I, FN, E-Cad, and α-SMA was used to assess fibrosis and EMT. **(B)** Semiquantitative analysis of E-Cad, α-SMA, FN, and COL I immunofluorescence staining of WT and KO RTE cells following hypoxia-reoxygenation injury. **(C)** Expression of profibrotic factors and proinflammatory cytokines following hypoxia-reoxygenation injury. Magnification: 200×. Data are shown as the means ± SD and were analyzed using the Student’s *t*-test. *P < 0.05, **P < 0.01, ***P < 0.001. WT, wild-type; KO, knockout; E-Cad, E-cadherin; α-SMA, α-smooth muscle; FN, fibronectin; COL I, collagen I.

Renal tubular epithelial cells constitute an important source of fibrosis and EMT. The expressions of TLR-9 post-IRI were also observed in this assay. TLR-9 was barely detectable in the sham group, whereas its expression increased considerably on days 1 and 2 post-bIRI and was mainly located on RTE cells. The expression of TLR-9 was rarely detected on day 7 or day 14 post-bIRI, while it was detected 28 days post-bIRI (Supplementary Figure 3). To further determine the roles of TLR-9 on RTE cells, RTE cells were isolated from WT and KO mice. Cellular immunofluorescence was performed to analyze the extent of fibrosis (as determined using COL I and FN) along with EMT (*via* E-Cad and α-SMA) of RTE cells under hypoxia-reoxygenation conditions. TLR-9 deficiency in RTE cells resulted in higher expression of COL I, FN, and α-SMA, albeit lower E-Cad levels under hypoxia-reoxygenation status (Figures 7A,B) (*P* < 0.01). Different reoxygenation durations were also explored in this assay; it was revealed that RTE cells suffered more severe injury upon 1 h of reoxygenation than at 24 h (data not shown). Profibrotic factor and proinflammatory cytokine production were also explored. Consistent with the results of immunofluorescence, gene expression of the profibrotic factor (TGF-β) in addition to proinflammatory cytokines (IL-1β, IL-6, and TNF-α) was higher in TLR-9^–/–^ than WT RTE cells (Figure 7C) (*P* < 0.01).

## Discussion

Ischemia-reperfusion injury constitutes the major cause of AKI and therefore leads to CKD (Jang and Rabb, 2015; Forni et al., 2017). In the present study, the roles of TLR-9 in AKI and CKD were explored using *in vivo* and *in vitro* models. Expressions of TLR-9 could be detected not only during AKI but also during CKD. No significant effect of TLR-9 was observed on AKI, while depletion of TLR-9 attenuated renal fibrosis after IRI. Depletion of TLR-9 decreased the intrarenal infiltration of macrophages, especially the M-2-like phenotype, and reduced the severity of inflammation during AKI→CKD transition *in vivo*.

Leukocyte infiltration serves to prime inflammation and fibrosis, thereby playing a fundamental role in their development (Tang et al., 2019). Naïve immune cells, especially macrophages, substantively contribute to non-infectious disease, including IRI. In a mouse model of doxorubicin-induced nephropathy, intravenous infusion of macrophages preactivated with a TLR-9 agonist could exaggerate renal injury (Wang et al., 2008). Alternatively, in a cisplatin-induced murine AKI model, depletion of TLR-9 in regulatory T cells resulted in more severe AKI (Alikhan et al., 2016). The effect of TLR-9, therefore, appears to depend on the specific cell type analyzed, with multiple kinds of cells able to be activated. This study also provides evidence for the roles of M2 macrophages in fibrosis. Although M2 anti-inflammatory cells contribute to injury repair, the CD206+ subset of M2 macrophages could aggravate renal fibrosis through multiple pathways, including MMT and TGF β1-Smad3 signaling. In this assay, MMT was also detected when rare cells that expressed both F4/80 and α-SMA were found; this suggests that the KO of TLR-9 decreased the infiltration of M2-like macrophages but had no effects on MMT.

In addition to leukocyte infiltration, proinflammatory and profibrotic cytokines could also accelerate the progression of fibrosis (Humphreys, 2018). Activation of TLR-9 can induce several signaling pathways. Moreover, proinflammatory and profibrotic cytokines, including IL-1β, IL-6, TNF-α, and TGF-β, could also be released (O’Keeffe et al., 2005; Kuo et al., 2013). Previous studies have shown that inflammation activation could directly contribute to fibroblast activation and renal fibrosis (Inoue et al., 2010); these results were also confirmed in the present study. In addition, as proinflammatory and profibrotic cytokines could also activate RTE cells as well as myofibroblasts, they may also contribute to inflammation and fibrosis. The effects of TLR-9 on these inflammation and fibrosis factors were also verified in the present study. Specifically, consistent with histology and immunohistology results, we found that inflammation and fibrosis signaling pathways were inhibited by the depletion of TLR-9.

Renal tubular epithelial cells were shown not only to contribute to the aggravation of inflammation but also to take part in EMT (Grande et al., 2015; Lovisa et al., 2015). Polymorphisms of TLR-9 have been confirmed to be associated with CKD in the Han Chinese population. Therefore, TLR-9 may play a critical role in the development of CKD (Yang et al., 2013). In the present study, the effects of global KO of TLR-9 were evaluated, such that all cell types in the mice exhibited TLR-9 depletion. The associated RTE cells were thus isolated to analyze the effects of TLR-9 depletion *in vitro*. Notably, TLR-9 deficiency did not result in better preservation of RTE cells under hypoxia-reoxygenation conditions; rather, fibrosis and EMT in TLR-9 KO RTE cells were indicative of more severe injury. The *in vitro* results were, therefore, in stark contrast with those from a previous report by Han et al. (2018) which indicated that TLR-9 activation on RTE cells promoted cell injury and death. One major difference between the two studies is the treatment of the RTE cells. Han et al. (2018) utilized a TLR-9 agonist to explore the effects of TLR-9 activation on RTE cells, whereas, in the present study, RTE cells were exposed to hypoxia-reoxygenation to evaluate the effects of TLR-9 or its depletion against injury. Another important difference between the two studies is that the RTE cells isolated by Han et al. (2018) appear to be random primary cultures used within 2–3 passages, while RTE cells used in this study were high-purity renal epithelial cells. Different cell cultures could also lead to different effects. We suggest that overactivation of TLR-9 on RTE cells may contribute to severe injury, whereas its depletion could also lead to fibrosis and EMT. These pieces of evidence also indicated that the therapeutic effects of TLR-9 inhibition on fibrosis *in vivo* mainly rely on the inhibition of macrophage infiltration and inflammation response.

Multiple cellular and molecular events are involved in inflammation and fibrosis of the kidney, including immune cells, RTE cells, endothelial cells, and the matrix components produced by these cells. The effects of TLR-9, thus, depend on the cells targeted in addition to the microenvironment. Although depletion of TLR-9 could result in the dysfunction of macrophages and other immune cells, it could also aggravate the injury to RTE cells during AKI. This may be why no significant difference was found regarding TLR-9 presence in AKI. In comparison, upon AKI→CKD transition, changes in the microenvironment may, in turn, alter TLR-9 function. Following reperfusion of blood and oxygen, TLR-9 is no longer necessary for the protection of RTE cells. In contrast, over-activation of TLR-9 not only stimulates immune cell-mediated inflammation but also promotes injury of renal intrinsic cells. Therefore, depletion of TLR-9 would serve to attenuate renal inflammation and fibrosis after injury. This model is also consistent with the expression of TLR-9 in the kidney post-bIRI, which decreased concomitantly with kidney repair, whereas continuous injury of the kidney led to TLR-9 accumulation.

This study has a few limitations. First, although a global KO mouse could be used to further analyze the mechanisms of TLR-9 in AKI→CKD transition, a conditioned KO mouse may be better for studying the detailed underlying TLR-9 mechanisms in specific cells. Second, although the effects of TLR-9 in RTE cells were explored in this assay, detailed mechanisms, especially that of the regeneration ability of RTE cells, should be further explored. Third, we did not rule out the effects of resident renal macrophages, which could also harbor a relevant population post-ischemic insult. Considering the reasons why mechanisms of AKI→CKD transition are complicated, further studies are needed to investigate the effects of TLR-9 on immune cells, especially macrophages.

Collectively, the results of this study provide novel insight regarding the roles of TLR-9 in acute and chronic kidney injury. Activation of TLR-9 post-AKI could lead to CKD development *via* the promotion of leukocyte infiltrations, interstitial inflammation, and EMT. Therefore, inhibition of TLR-9 may provide a new therapeutic option for the prevention of AKI→CKD transition.

## Data Availability Statement

The original contributions presented in the study are included in the article/Supplementary Material, further inquiries can be directed to the corresponding author/s.

## Ethics Statement

The animal study was reviewed and approved by the Institutional Animal Care and Use Committee of Sun Yat-sen University. Written informed consent was obtained from the owners for the participation of their animals in this study.

## Author Contributions

HZ and QS conceived and designed the experiments. HZ, YZ, and LL performed the experiments with the help of JH, ZY, ZL, RZ, and YY. HZ and YZ analyzed the data and drafted the manuscript. All authors helped to interpret results and approved the final version of the manuscript.

## Conflict of Interest

The authors declare that the research was conducted in the absence of any commercial or financial relationships that could be construed as a potential conflict of interest.
