# Supplementary material for: Depletion of Toll-Like Receptor-9 Attenuates Renal Tubulointerstitial Fibrosis After Ischemia-Reperfusion Injury
Source: Front Cell Dev Biol. 2021 Feb 12;9:641527. doi: 10.3389/fcell.2021.641527 (PMC7907438; doi:10.3389/fcell.2021.641527)
Supplement: Supplementary file 1 [file Table_1.DOCX]

**Supplementary material**

## Depletion of Toll Like Receptor-9 Attenuate Renal Tubulointerstitial Fibrosis after Ischemia-Reperfusion Injury

Haofeng Zheng^#^, Yannan Zhang^#^, Lei Li^#^, Rui Zhang, Zihuan Luo, Zhe Yang, Yongrong Ye, Jiannan He, MD, and Qiquan Sun, MD, PhD

Organ Transplantation Research Institute of Sun Yat-sen University, the Third Affiliated Hospital of Sun Yat-sen University, Guangzhou, China

**Brief title**: Critical Roles of TLR-9 in Renal Fibrosis

**Correspondence author:** Dr Qiquan Sun, 2693, Kaichuang Avenue, Huangpu District, Guangzhou, Guangdong, China, 510630, Email: [sunqiq@mail.sysu.edu.cn](mailto:sunqiq@mail.sysu.edu.cn); Tel. +86 20 82179737; Fax. +86 20 82179739

^#^These authors contributed equally to this work.

**This article contains the following supplemental material online at:**

**
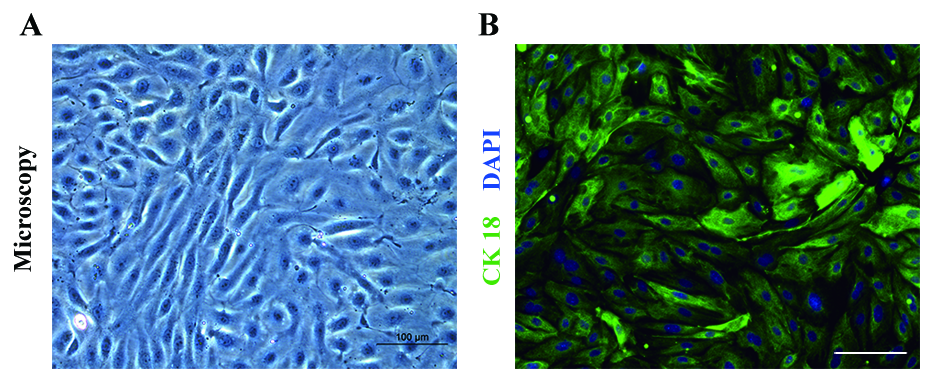
**

**Supplemental Figure 1. Verification of RTECs in vitro.** RTE cells were isolated and immunofluorescence of CK18 was used to verify the phenotype of epithelial cells. Results were acquired from three independent assays. (A) Light microscopy image of RTECs. (B) Immunofluorescence of CK18. Magnification: 200x. RTE cells: renal tubular epithelial cells; CK18: Cytokeratin 18.


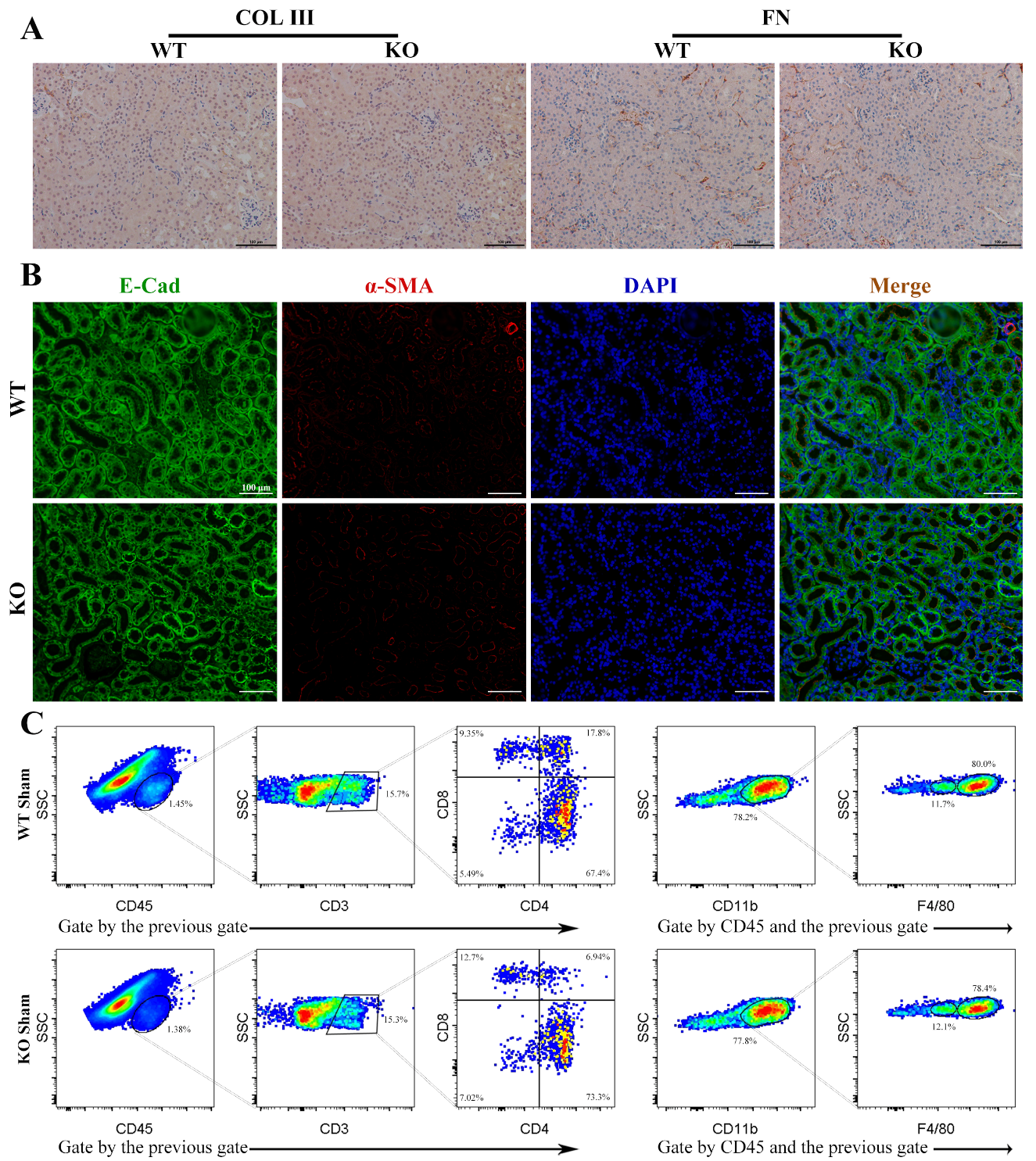


**Supplemental Figure 2.TLR-9 depletion has no obvious effect regarding fibrosis or number of intrarenal leukocytes between WT sham and KO sham group.** (A) Representative COL Ⅲ and FN staining immunohistochemistry in renal cortical sections from WT sham group and KO sham group; (B) Representative fluorescence microscope images of E-Cad (Green) and α-SMA (Red) staining of the kidneys of WT sham group and KO sham group; (C) Representative dot plots of CD45+ cells and different types of leukocytes (T cells and macrophages) of WT sham group and KO sham group.


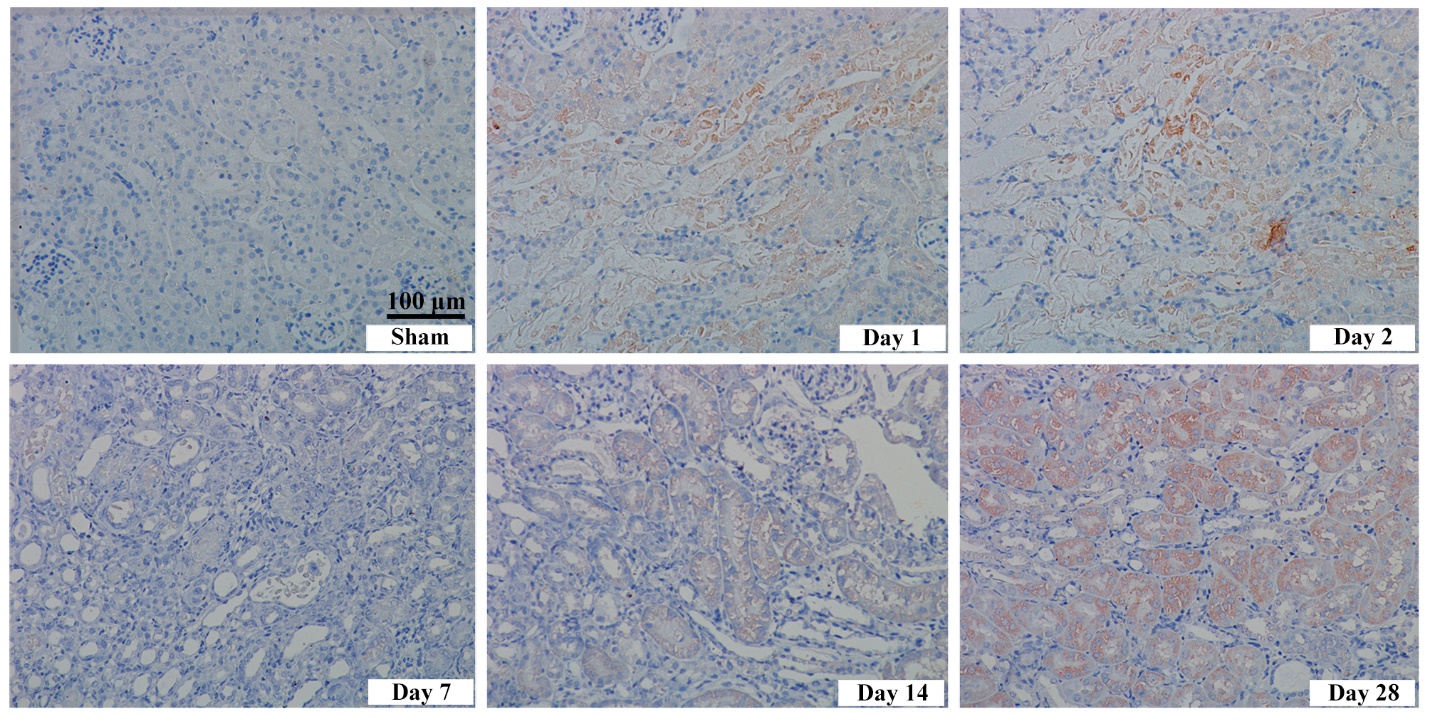


**Supplemental Figure 3. Expressions of TLR-9 in the kidney following IRI.** Immunohistochemistry of TLR-9 in the kidney following IRI or in the Sham group. Kidneys were collected at 1, 2, 7, 14, and 28 days after reperfusion. Tissue sections are representative of five mice per group. Magnification: 200x. TLR-9: toll like receptor-9; IRI, ischemia–reperfusion injury.
